# Effect of Recycling and UV Ageing on the Properties of PLA-Based Materials Used in Additive Manufacturing

**DOI:** 10.3390/polym17131862

**Published:** 2025-07-03

**Authors:** Petr Jirků, Miroslav Muller, Rajesh Kumar Mishra, Jaroslava Svobodová

**Affiliations:** 1 Department of Material Science and Manufacturing Technology, Faculty of Engineering, Czech University of Life Sciences Prague, Kamycka 129, 165 00 Prague, Czech Republic; jirkup@tf.czu.cz (P.J.); muller@tf.czu.cz (M.M.); svobodovajaroslava@tf.czu.cz (J.S.); 2 Faculty of Mechanical Engineering, J. E. Purkyně University in Ústí nad Labem, 400 03 Usti nad Labem, Czech Republic

**Keywords:** polylactic acid (PLA), coffee ground (CG), biocomposites, UV ageing, mechanical properties, photodegradation, material recycling, 3D printing

## Abstract

This article focuses on the possibility of using biodegradable polymer-composite materials in additive manufacturing via fused deposition modelling (FDM) 3D printing. The main objective was to experimentally verify the technical feasibility of the repeated use of recycled PLA and PLA composites containing 10% natural coffee-ground (CG) filler in a print–degradation–recycling–print cycle. Special attention was paid to simulated ultraviolet radiation as a degradation factor affecting the materials’ mechanical properties. Pure PLA and PLA_CG were compared at four levels of degradation time and after subsequent recycling. The results show that the inclusion of coffee-ground filler slightly reduces the initial strength but enhances the 3D-printed material’s resistance to UV degradation and thus extends its functional service life. Unlike pure PLA, which loses its processability after 12 weeks, PLA_CG retains structural integrity and mechanical functionality. The research confirms the potential of recycled PLA composites with natural fillers for sustainable manufacturing and supports their use within a circular economy framework.

## 1. Introduction

In recent decades, there have been significant developments in the field of additive manufacturing [1,2]. As far as fused deposition modelling (FDM) 3D printing technology is concerned, there has been a significant expansion of this additive manufacturing method, especially in the fields of leisure and domestic appliances. It has become one of the most widely used 3D printing technologies due to its wider availability, ease of application, and relatively lower acquisition costs [1,3,4]. In recent years, additive technologies have also found applications in the engineering industry, where they are increasingly being integrated into various production processes, especially in the field of prototyping, the production of functional components, and spare parts, with the aim of reducing inventory and downtime in the event of a failure. In cases where the properties of polymeric materials used in additive manufacturing are not sufficient for direct use in industry, 3D printing technology has found application at least as an effective tool for the rapid and affordable production of auxiliary fixtures for marking and workplace layout in accordance with the 5S method [5]. This approach facilitates the activities of assembly workers and at the same time contributes to the unification and streamlining of production processes [6,7]. Additive technologies are distinguished by their flexibility and production speed in the case of specific fixtures and holders.

There is a growing interdependence between the development of additive technologies, materials engineering, advanced digital design tools [8,9], and composite materials, offering a wide range of applications [10,11]. The functionality, versatility, and mechanical durability of 3D printed products are continuously enhanced by the use of new materials such as metals, polymer composites, or other highly specialised products. At the same time, a significant shift is taking place in the field of 3D printing devices themselves, where the implementation of artificial intelligence (AI) and machine learning (ML) elements is enabling the optimisation of production processes, more efficient use of materials, and the minimisation of waste material through continuous process control [12,13,14,15,16,17].

Although some polymeric materials are proven to be compostable, their compostability may not be entirely clear under the European Union (EU) Regulation 2023/2055 of 25 September 2023. The Regulation points out that, even with biodegradation, persistent residues of synthetic polymer microparticles may remain in the environment that do not meet the criteria for full biodegradation [18]. This poses a potential environmental risk, particularly when these materials enter industrial or domestic composting systems.

The EU Regulation also stresses that the label ‘compostable’ alone does not mean that microplastics will not be released into the environment, especially if the fixed conditions for composting (e.g., time, temperature, microbial activity) are not ensured [19]. For this reason, it is advisable to handle compostable polymers with higher levels of caution and to assess their environmental impact comprehensively, not just based on marketing propaganda.

Recycling of waste PLA is one approach to reducing the environmental burden and at the same time optimising the utilisation of material resources, especially by reducing the need for virgin PLA [20,21,22,23,24]. The application of recycled PLA (rPLA) can contribute not only to cost savings in material, but also to the reduction of energy requirements associated with the production and processing of the primary polymer product [25]. This approach thus appears to be an economically viable alternative that has the potential to reduce the overall cost of producing 3D printing filaments and subsequently the final products [26,27]. FDM 3D printing technology offers the possibility of using rPLA, but its practical application is still limited by several technological obstacles. One of the main challenges is the degradation of polymer chains during repeated thermal and mechanical processing, which leads to a reduction in chain length and consequently deteriorates the mechanical properties of the 3D-printed products. Furthermore, there are changes in the crystalline filament, increased shrinkage during printing due to the modified melting temperature, and an overall decrease in the durability of the products [28,29,30]. These factors not only have technological implications but also can affect the economic and environmental aspects of the production process.

Successful implementation of recycled PLA requires the active cooperation of a wide range of stakeholders, from material and printing equipment manufacturers to the users themselves. There is a growing interest in FDM printing technology, and there is a considerably higher number of studies on the use of PLA [31,32,33], the optimisation of print parameters [34,35,36], recycling of thermoplastics [33,37,38,39,40], and the sustainability of additive manufacturing [41,42,43]. However, there is still a lack of comprehensive and systematic analysis focused exclusively on the use of recycled PLA in this method. There is almost no research reported that focuses on PLA waste treatment methods, preparation techniques for recycled PLA filaments, and their specific impact on the properties of final 3D-printed products. Also, the environmental and socioeconomic impacts associated with the wider application of recycled PLA in additive manufacturing are still not properly understood.

In recent years, there has been a growing interest in incorporating bio-based waste materials into polymer matrices in order to enhance their environmental profile and support circular economy strategies. One such material is waste coffee grounds (CG), which are generated in large volumes globally and often end up as an organic waste material. Their use as a filler in PLA is motivated by multiple factors. Firstly, CGs are low-cost, renewable, and biodegradable, making them an attractive alternative to conventional mineral or synthetic fillers. Secondly, they contain functional groups such as hydroxyl and carboxyl groups, which can interact effectively with the polymer matrix. Furthermore, CGs contain bioactive components, including polyphenols, melanoidins, and natural pigments, which may provide antioxidant and UV-stabilising effects when embedded in polymer systems. The typical chemical composition of waste coffee grounds includes approximately 30–35 wt.% hemicellulose, 20–27 wt.% lignin, 8–15 wt.% cellulose, 13–17 wt.% proteins, 10–15 wt.% lipids, and minor fractions of polyphenolic compounds and minerals [44]. These constituents contribute to the filler’s potential to enhance resistance to UV-induced degradation and modify mechanical behaviour through increased light absorption, free radical scavenging, and potential barrier effects. For this reason, CG was selected as a promising filler in the present study.

The aim of the research was to verify the technical feasibility of repeatedly using biodegradable PLA filled with coffee grounds (CG) in additive manufacturing via the FDM process. The study focused on evaluating changes in mechanical properties under combined UV-induced degradation. It tracked differences between neat PLA and PLA containing 10% CG at each stage of the material life cycle. The outcome is an assessment of the application limits of these materials in terms of functional service life and sustainability. As such, the research advances circular economy approaches in the realm of 3D printing.

## 2. Materials and Methods

Based on the analysis of secondary sources and the literature, PLA material and a composite variant of PLA material with coffee-ground fillers were selected for the research. In the experimental part, emphasis was placed on the preparation of samples of pure PLA and its composite system with natural coffee-ground fillers. The natural reinforcement underwent rigorous pre-treatment, which included several technical steps before application to the polymer matrix. A Retsch MM 400 oscillating mill (Retsch GmbH, Munich, Germany) was used to grind the raw coffee grounds to achieve the desired particle size suitable for use in additive technologies. Subsequently, fractional analysis was performed using Haver EML vibrating screens (Haver & Boecker OHG, Oelde, Germany). To remove residual moisture from the natural filler and PLA granulate, the materials were dried in a Memmert UN30 laboratory dryer (Memmert GmbH + Co. KG, Schwabach, Germany). The coffee-ground fillers were dried at 90 °C for 48 h. They were then separated into several fractions using a HAVER EML digital plus sieving machine, Haver & Boecker OHG, Oelde, Germany). A particle size range of 0.2–0.3 mm was chosen for this research, as for additive FDM technology, a nozzle size of 0.4 mm is usually used [45].

PLA INZEA F2 HTS 451 with 10 wt.% coffee ground was chosen as the polymer matrix of the composite.

The PLA polymer was dried in a laboratory oven at 45 °C for 6 h. The prepared filler and matrix were subsequently processed in cooperation with the Technical University of Liberec on a Collin ZK 25E twin-screw granulation line (COLLIN Lab & Pilot Solutions GmbH, Maitenbeth, Germany) equipped with an ECON EWA 10 granulation unit (ECON GmbH, Weisskirchen, Austria). The Collin compounding unit is equipped with a twin-screw extruder, where the screws are divided into several sections (conveying, kneading, and mixing zones). The screws themselves have a diameter of 25 mm and a length of 36 mm. The compounding principle involved placing PLA granulates with coffee grounds into the main hopper, from which this mixture was removed and then melted by friction and heat. After melting, the molten mixture was homogenised and then fed into the extrusion head of the machine. A knife head of the granulation unit was connected to the extrusion head, where the material was cut. Cold granulation was chosen for the experimental conditions. In this method, the cut granulates were cooled by flowing water, which was then carried to a section where the water was separated from the polymeric material. The polymeric material was then carried by a stream of air to a cyclone, where it was dried by the air stream, and the residual heat was removed. Further, it was poured into containers for extruding into filaments (as the material was cooled with water, it was subsequently dried at a temperature of 50 °C for 6 h before processing it into filaments). The screw speed was set to 150 rpm, and the knife head speed was 3000 rpm. The temperature of the preheated hopper was set at 40 °C. The individual temperature zones of the compounding unit were set as follows: Zone 6: 135 °C, Zone 5: 145 °C, Zone: 4: 150 °C, Zone 3: 145 °C, Zone 2: 146 °C, Zone 1: 145 °C, extruder 150 °C. Zone 1 was closest to the extruder, while zone 6 was closest to the hopper. These composite granulates served as the starting material for further processing.

The sample sets for material testing and controlled UV degradation were made of pure PLA granulate and composite granulate with 10% coffee grounds. For the samples subjected to UV degradation, recycling, i.e., crushing of the test samples, extrusion into the shape of a printing string and re-creation of the test bodies using additive FDM technology, was carried out. Prior to artificial ageing and mechanical testing, all samples were stored in re-sealable polyethylene bags together with silica gel desiccant to minimise moisture uptake. To prevent unintended photo-degradation, the sealed bags were placed in an opaque black plastic box and stored under laboratory conditions at (23 ± 2) °C and (50 ± 5) % relative humidity. A summary of the test samples is given in Table 1:

After creating test bodies according to EN ISO 527-2 [46], degradation was performed in a UV chamber of the Q-SUN Xenon Test Chamber Model Xe-3-HS (Q-Lab Corporation, Westlake, OH, USA). The device allows for simulating UV-B radiation with a wavelength of 340 nm, which best replicates solar radiation in outdoor conditions. The test samples were exposed to UV radiation under simultaneous exposure to elevated humidity and temperature in the homogenised environment of the laboratory simulator. The facility contains three xenon lamps for radiation simulations. A black temperature control panel is installed inside the chamber, the temperature of which is adjustable on the display of the degradation device. The chamber temperature was set at 40 °C, the black panel temperature at 55 °C, and the relative humidity inside the chamber at 40%. The power of each lamp was 0.55 W/m^2^, and the ultraviolet wavelength was 340 nm. The degradation was divided into several groups according to the time of exposure to UV degradation

The created sets of samples, as shown in Table 1, were degraded for 4 weeks and 12 weeks. The samples were inverted at weekly intervals during degradation to ensure that degradation occurred evenly on both sides of the test bodies.

The sets of test bodies were then subjected to mechanical testing on a LABTest 5.5 ST universal machine with an AST KAF 50 kN measuring unit. This was a static tensile test at a speed of 10 mm·min^−1^. The standard test method, EN ISO 527-2 [46] Plastics—Determination of tensile properties was used.

**Figure 1:**
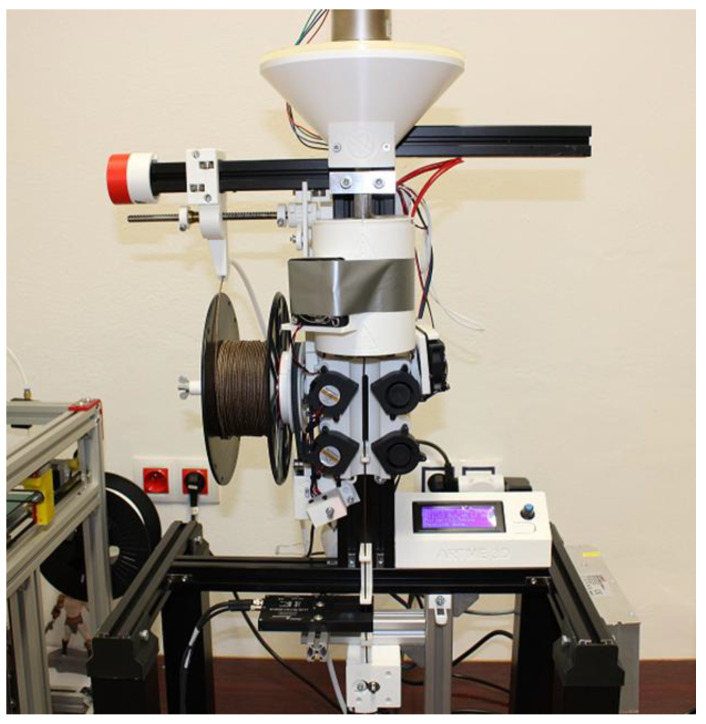
Composite filament extrusion.

For filament production, a benchtop extruder MK2 from ARTME 3D (Artme GmbH, Waldsee, Germany) with laser diameter measurement using L-LAS-TB-F-6x1-20/40-AL (Sensor Instruments Entwicklungs-und Vertriebs GmbH, Thurmansbang, Germany) was used. The melting temperature of the material was set to 172 °C. Figure 1 shows the manufacturing process of the PLA_CG composite filament.

A single-screw extruder with a single heating zone was employed for filament fabrication. The barrel temperature was kept identical for all materials. The screw length was 215 mm, with an active processing section of ≈150 mm. At 7 rpm, the residence time in the heated zone was ~5 min, after which the melt was extruded and cooled by axial fans operating at maximum speed to ensure uniform cooling.

Filaments with a standardised diameter of 1.75 mm were created from pure PLA and PLA reinforced with coffee-ground fillers, suitable for subsequent 3D printing of samples and their material testing.

Two types of filaments were produced. The first was made from pure PLA INZEA F2 HTS 451 granulate, the second variant contained 10 wt.% coffee grounds with an average filler size of 0.2–0.3 mm. The PLA granulate used from NUREL BIOPOLYMERS has inherently optimised properties for extrusion and use in additive technologies.

**Figure 2:**
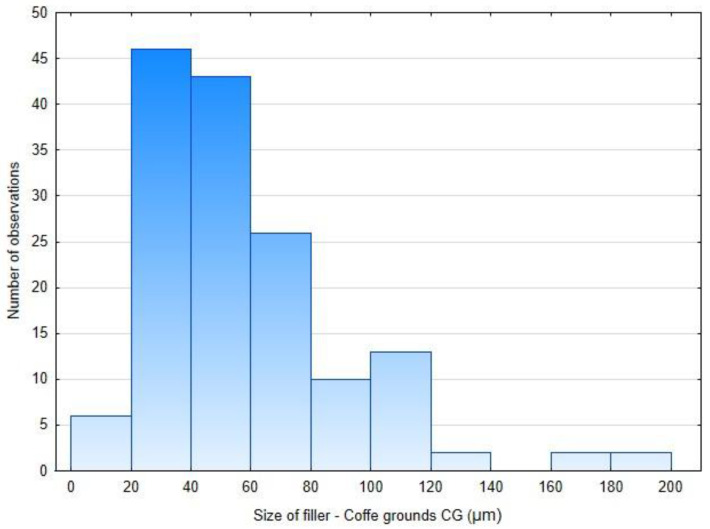
Frequency histogram of size of CG used as filler in PLA materials.

The particle size of the coffee-ground (CG) filler was determined by optical analysis of scanning electron microscopy (SEM) images using (Gwyddion software, version 2.68, Brno, Czech Republic). The fractional size distribution of the CG particles is shown in Figure 2. The results reveal that particles in the range of 20–80 µm predominated in the samples.

**Figure 3:**
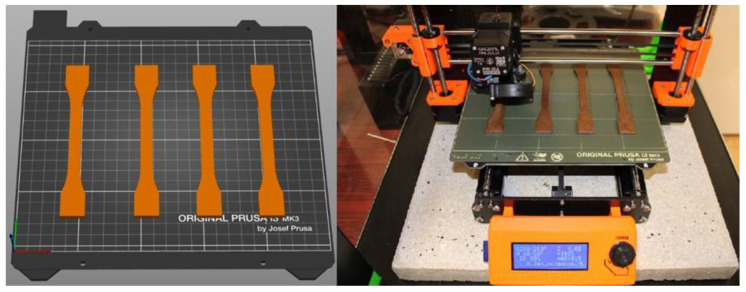
PrusaSlicer print setup on the left, sequential 3D printing of samples on the right.

The filaments were then used to create test bodies using additive technology on a Prusa i3 MK3S 3D printer, which is shown in Figure 3. The printing parameters set in the (Prusa Slicer software version 2025, Prusa Research, Holešovice, Czech Republic) are shown in Table 2.

Sequential printing was chosen for printing the samples because of the susceptibility to stringing in filaments with natural filler. Stringing (or oozing) in FDM 3D printing refers to the unwanted formation of thin filaments of base material that are pulled out of the printing nozzle as the print head moves between two models. In Figure 3, the left side shows the preparation of the models in Prusa Slicer, while the right side shows the printed composite samples on the Prusa 3D printer, where the three test bodies on the right are already created, and the printer is finishing the last test body on the left.

A Prusa i3 MK3S 3D printer from (Prusa Slicer software version 2025, Prusa Research, Holešovice, Czech Republic) was used to produce test specimens for the evaluation of mechanical properties. The tensile test was carried out in accordance with the ISO 527-2 standard [46] (Plastics—Determination of tensile properties—Part 2: Test conditions for moulding and extrusion plastics).

**Figure 4:**
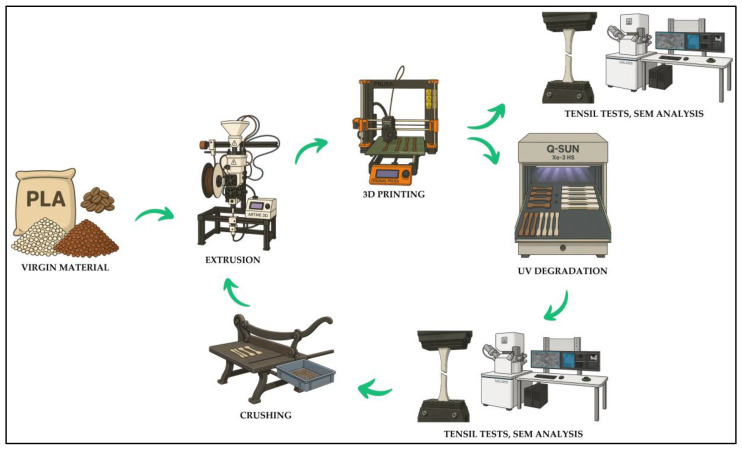
Graphical representation of the sample production process.

The selected sets were then subjected to mechanical tests, crushed, extruded into filament form and reworked into test bodies by additive manufacturing. These were then tested on the LABTest 5.50 ST (LABORTECH Ltd., Brno, Czech Republic) with the AST KAF 50 kN measuring unit (AST Angewandte System Technik GmbH, Dresden, Germany) and evaluation software (Test&Motion LABORTECH Ltd., Brno, Czech Republic). The entire production process is shown graphically in Figure 4.

**Figure 5:**
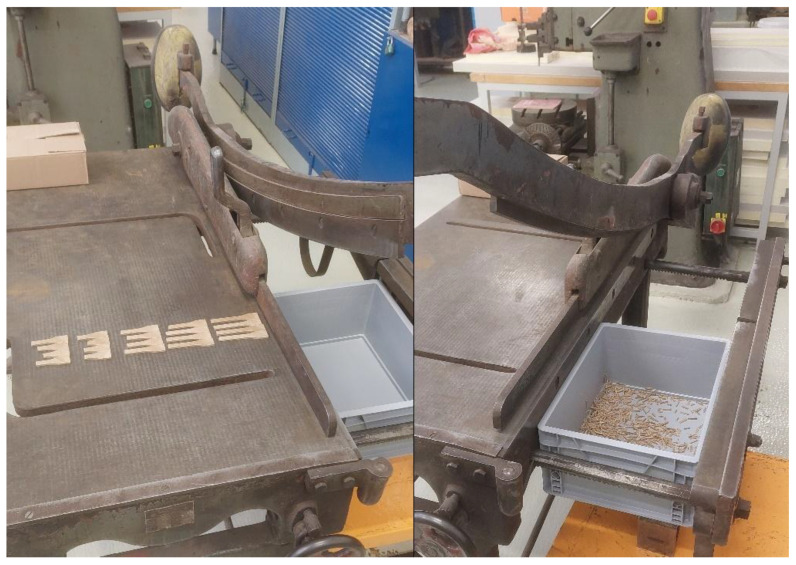
Crushing of samples.

Crushing after mechanical testing is shown in Figure 5. After mechanical testing, the degraded and tested samples were crushed for insertion into the extruder. The material thus treated was then re-extruded to produce test specimens for static tensile testing.

In order to avoid the thermal influence of the samples during grinding by high-speed mills, a manual grinding method using a shearing mechanism was chosen, where high temperature rise due to friction is avoided.

**Figure 6:**
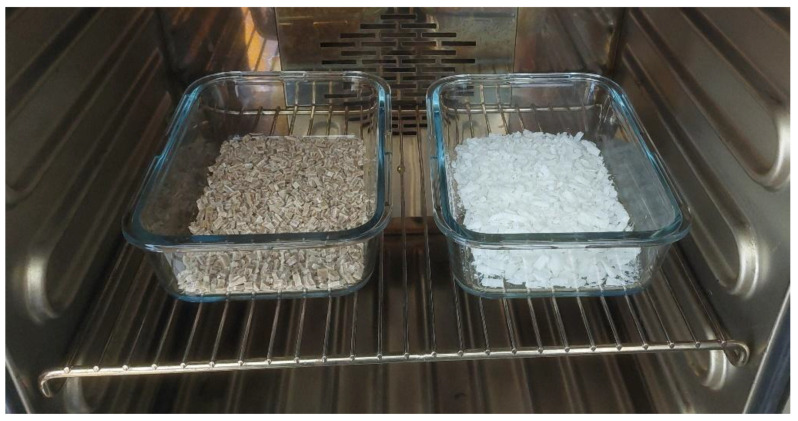
Drying of crushed material for recycling.

Due to the size of the auger inlet opening of the MK2 benchtop extruder, a size fraction of 4 mm of crushed material was selected. Additive technology was used to screen the required fraction. A model was created, and a sieve was printed with the required aperture size, i.e., 4 mm × 4 mm. This is proof of how fast and efficient the use of additive manufacturing can be in practice. The crushed, sieved material was then dried at 50 °C for 8 h, an example of which is shown in Figure 6. These materials were then used to form test bodies for further mechanical testing. The batches of specimens thus formed were designated as recycled in Table 1 and Table 3.

**Figure 7:**
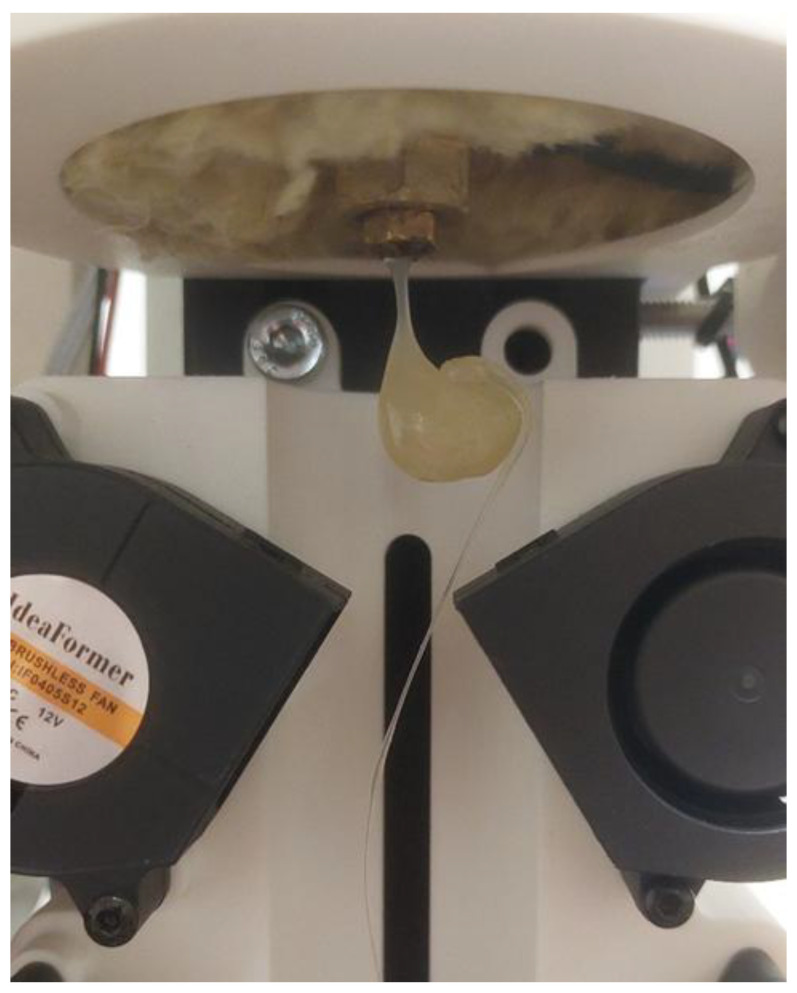
Extrusion of excessively degraded PLA material.

Samples formed from pure PLA that were exposed to degradation for 12 weeks experienced a high rate of degradation; the material became very brittle, flowed out of the extruder during processing, and behaved inconsistently. This material could not be further processed; therefore, the tensile strength and displacement test results for Variant 9 are not shown in Table 3. An example of the extrusion behaviour of the degraded PLA material is shown in Figure 7. The change in colour of the material (yellowing) as compared to the originally clear PLA material can be observed.

**Figure 8:**
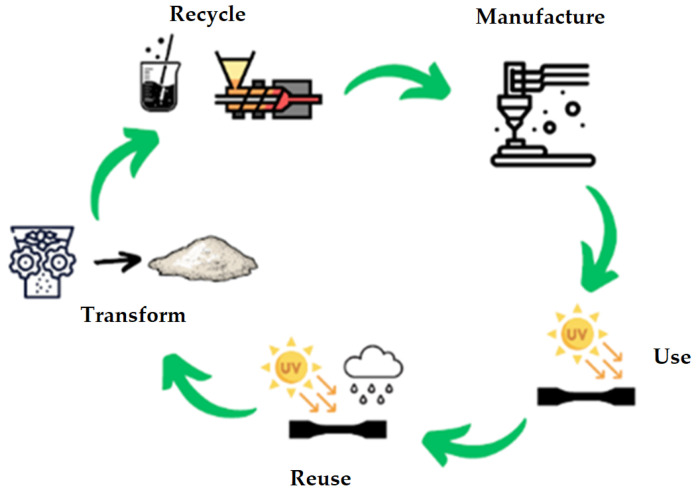
The basic premise of the circular economy.

All these steps are simply described in the diagram of the basic premise of the circular economy in Figure 8. A filament was created, from which samples were subsequently formed by additive technology. These samples were degraded in the UV chamber that simulated long-term exposure to sunlight. The degraded samples were tested and again transformed into filament and then into test samples. All this was done to test the possibilities and limits of recycling, which is undoubtedly one of the main objectives of the circular economy in the field of materials used for additive manufacturing.

The interactions at the interfaces of the fillers with the polymer matrix, as well as the microstructural arrangement of the filler materials, were investigated in detail using scanning electron microscopy (SEM), which allowed the identification of morphological changes and the assessment of the cohesion of the individual phases of the composite.

**Figure 9:**
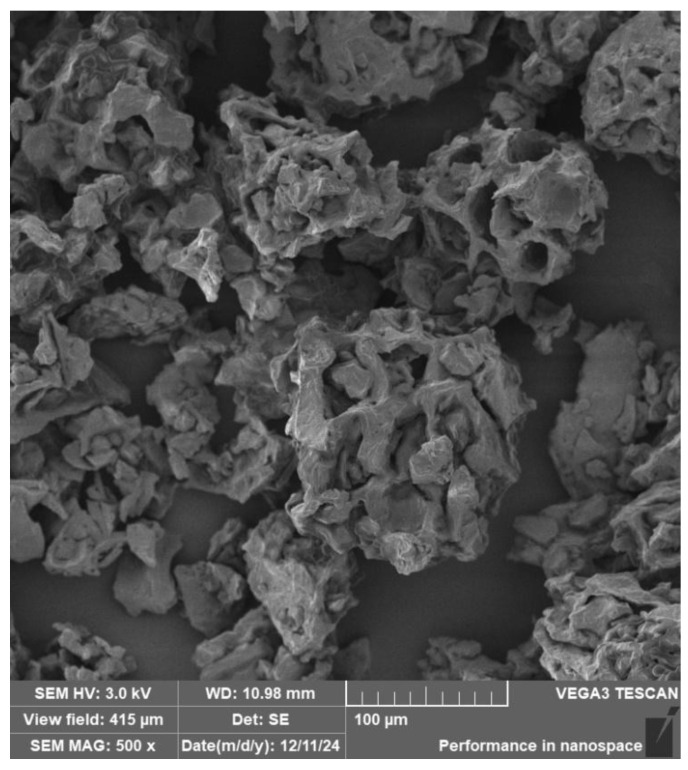
SEM image of coffee grounds (MAG 500×).

An image of the coffee grounds taken with a scanning electron microscope (SEM), in this case at 500× magnification, can be seen in Figure 9.

The samples for analysis using scanning electron microscopy (SEM) were prepared using the standard method. In the first step, the fractured samples after the static tensile test were sorted on a metallographic saw, Accutom-10/-100 (Struers GmbH, Willich, Germany). Then, the samples were cut without the supply of any cooling liquid to avoid contamination of the fracture surface with the cooling medium. The division occurred at a distance of approximately two centimetres from the fracture surface so as not to affect and damage the surface profile by cutting. After removal from the metallographic saw, the samples were cleaned and blown with compressed air. The samples prepared this way were subsequently placed on prepared pins suitable for placement in the scanning electron microscope chamber. The pins were 12.5 mm in diameter, made of aluminium alloy, with a mandrel 3.2 mm in diameter and 8 mm in length. A 12.5 mm diameter adhesive carbon target was glued to the surface of the pin, onto which the sample was glued with the flat side after cutting (with the fracture surface facing upwards). This prepared the fracture surface for gold sputtering, a necessary step to bring the sample into a conductive state before SEM observation. The samples were placed on a Ø 50 mm rotation stage with a rotation speed of 8–20 rpm. A vacuum was created in the chamber of the machine and the sample surface was sputtered with a 10 nm thick gold layer using a Quorum Q150R ES Plus sputtering machine (Quorum Technologies–Judges House, Laughton, UK). The samples thus prepared were then inserted into the chamber of the electron microscope. SEM analysis was performed using a TESCAN VEGA 3 XMU (TESCAN ORSAY HOLDING a.s., Brno, Czech Republic). An acceleration voltage of 3 kV, SE (secondary electron) detector, and DEPTH imaging mode at different magnifications were chosen to observe the samples.

## 3. Results and Discussion

The test results are shown in Table 3. A static tensile test was performed at a test speed of 10 mm·min^−1^. The average values (10 specimens tested for each sample) of tensile strength and displacement of the specimen were then plotted in graphs to clearly illustrate the differences between the different samples.

**Figure 10:**
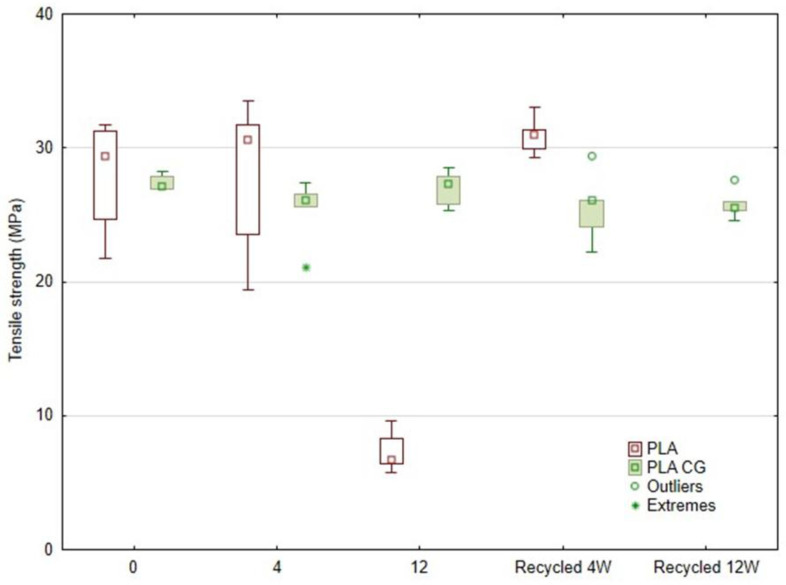
Statistical display of tensile strength results as a function of weeks of UV degradation and recycling.

Figure 10 presents a graph comparing the tensile strength of PLA and PLA_CG (PLA with coffee grounds) as a function of the duration of UV degradation and subsequent recycling (recycled after 4 weeks and recycled after 12 weeks). The results show that pure PLA exhibits the highest initial strength, approximately 28 MPa, with no significant decrease in mechanical properties after 4 weeks of UV exposure, but an increase in the variance of the results. However, after 12 weeks of UV degradation, there is a significant reduction in strength to below 10 MPa, indicating significant disruption of the polymer chain due to photo-degradation. The increased scatter of results may be promoted by the sample fabrication technology (FDM 3D printing), where the material is deposited in a layer-by-layer manner in which perfect bonding does not occur.

In the case of PLA_CG, a lower initial strength (approx. 26–28 MPa) is observed, but the values remain relatively stable even after prolonged UV exposure. After 12 weeks, the tensile strength is still around 24–25 MPa, indicating a higher resistance of this composite to UV degradation. This effect may be attributed to the presence of coffee grounds, which may act as a partial barrier against UV penetration or contribute to the absorption of free radicals generated during polymer degradation [47,48].

Interesting differences also appear in the recycled samples. Recycling PLA after 4 weeks of UV degradation does not lead to a deterioration in strength, which remains at the level of the non-degraded material. In contrast, recycling of pure PLA after 12 weeks was no longer possible, and the strength of the degraded samples was very low. In contrast, PLA_CG retains its properties after recycling even after 4 and 12 weeks of UV degradation, indicating a higher recyclability and stability of this composite material.

These results show that although PLA achieves higher initial strength, it is less resistant to environmental influences and subsequent recycling in the long term. PLA_CG, on the other hand, is a more stable material for specific applications where long-term stability and reusability are required, e.g., in the context of the circular economy concept.

**Figure 11:**
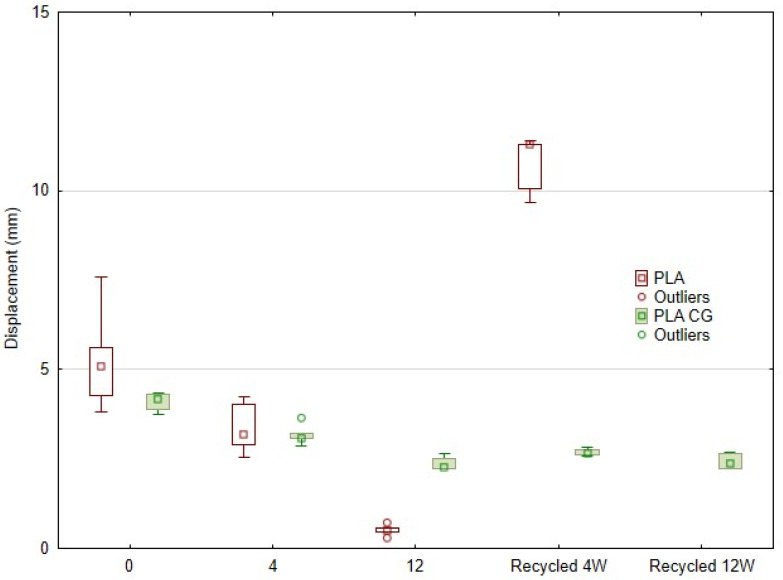
Statistical display of displacement results as a function of weeks of UV degradation and recycling.

Figure 11 presents a plot comparing the relative displacement versus the time of degradation and subsequent recycling of the PLA and PLA-CG composite samples. Experimental conditions included controlled degradation for 4 and 12 weeks and recycled samples after 4 and 12 weeks of degradation, designated Recycled 4W and Recycled 12W.

Based on the tensile test results, it was found that the addition of coffee grounds (CG) to the PLA matrix reduced both the tensile strength (σm) and displacement (ΔL) of the undegraded specimens (Variants 1 and 2). After 4 weeks of UV degradation, the PLA_CG material (variant 4) showed a 7.5% decrease in displacement. Interestingly, despite the simulated ageing in the UV chamber, the pure PLA (variant 3) is at a comparable level to the baseline, even showing a slight increase of 0.7% in the tensile strength.

After subsequent recycling (crushing, extrusion, and 3D printing of the degraded material), the pure PLA after recycling (variant 5) showed an increased tensile strength of 11.41% with respect to reference variant 1 and a significantly higher ductility (103.41% with respect to reference variant 1), which can be attributed to the possible remelting and rearrangement of the crystalline regions of the material. PLA_CG (variant 6) after recycling showed a decrease in ductility of 34.36% with respect to reference variant 2 and a simultaneous decrease in strength (by 6.75% with respect to reference variant 2), which may be related to possible degradation occurring in the natural filler component. This decrease subsequently stopped, and there was even a slight increase in tensile strength for variant 8, which was UV-degraded for 12 weeks, showing a 6.29% increase for variant 10 (which was recycled) with respect to variant 4, and a 1.82% increase for variant 8.

With long-term degradation (12 weeks), the pure PLA (variant 7) showed a significant decrease in both mechanical parameters studied. In contrast, PLA_CG (variant 8) retained a relatively high tensile strength (26.95 MPa) even after 12 weeks, indicating stabilisation against the UV degradation process. For the recycled specimens after 12 weeks of UV exposure (variant 10), the PLA_CG composite maintained stable values of maximum tensile strength (σm) and displacement (ΔL). This result confirms very good stability of the mechanical properties of the material and indicates its suitability for recyclability, even when exposed to UV radiation. As UV exposure is a common degradation factor for polymeric materials and a realistic scenario when using products created by additive technologies, this stability is significant.

**Figure 12:**
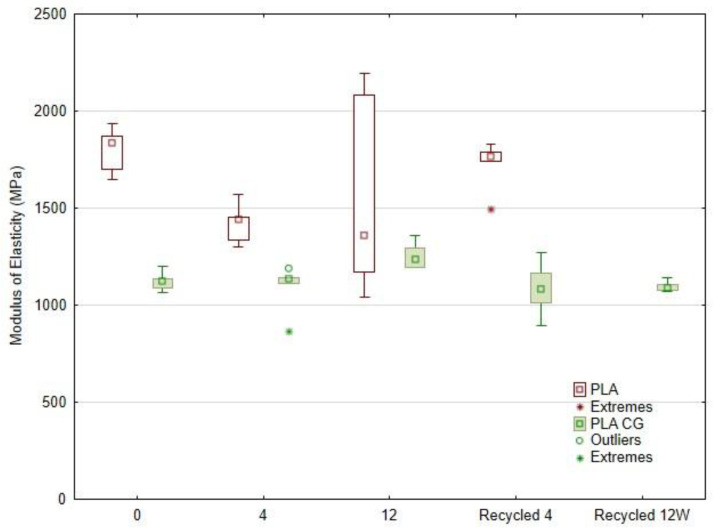
Young’s modulus of neat PLA and PLA-CG after UV ageing and recycling.

Figure 12 shows the Young’s modulus of the tested specimens. For neat PLA, the average modulus decreased by 20.9% after 4 weeks of UV exposure and by 12.6% after 12 weeks. After 12 weeks of degradation, the coefficient of variation rose to ≈35%, whereas in all other series it remained below 8%. Remarkably, re-extrusion and re-printing of the UV-aged PLA raised the modulus to 1 723.6 ± 133.0 MPa, almost the original value.

The increase in the elastic modulus of UV-degraded and subsequently re-extruded PLA using 3D printing can be explained by a combination of physicochemical phenomena. UV radiation can induce crosslinking of polymer chains and partial cleavage, which increases the degree of crystallinity and orientation of the chains during re-extrusion. Degradation residues are also removed, resulting in a cleaner and stiffer polymer matrix. This effect is confirmed by studies that report an improvement in the mechanical properties of re-extruded PLA after UV degradation due to a rearrangement of the structure and an increase in crystallinity [49,50,51].

Sasse et al. [49] found that re-extruded PLA exhibited a higher modulus in some cases due to increased crystallinity and chain orientation. UV ageing can induce structural changes in PLA which, when subsequently re-extruded during filament production, lead to increased crystallinity or purity and thus a higher modulus. It is therefore not a degradation in the classical sense but a restructuring of the polymer network [50].

Adding the coffee-ground filler (labelled CG) reduced the initial modulus by ≈38%. Studies have observed a decrease in initial modulus of up to 24% in case of unmodified coffee grounds in PLA, with similar reductions documented in other studies [52,53]. Hence, the filler substantially lowers the stiffness of virgin PLA after UV ageing, confirming trends reported for natural fillers [53].

Coffee ground (CG) is a predominantly amorphous, porous biomass with lower stiffness. Its addition to PLA in 3D printing leads to a reduction in modulus, mainly due to the lower stiffness of the filler itself. The inclusion of CG particles/fillers disrupts the continuity and cohesion of the PLA matrix, impairing stress transfer in the material. It introduces softer phases into the PLA matrix, disrupting the integrity and continuity of the polymer matrix. The weak adhesion between CG and PLA creates a potential failure-prone interface, which further reduces the mechanical resistance. This results in reduced resistance to deformation and a lower modulus of elasticity.

Nagengast et al. reported that DSC analysis (differential scanning calorimetry) reveals that re-granulation of UV-aged PLA often leads to an increase in crystallinity, which can improve the elastic modulus through improved chain ordering and thermal “reset” of the material [50]. De Bomfim et al. [54] also analysed the mechanical impact of adding CG to PLA. The crystallinity of PLA was found to have increased after processing and the addition of fillers due to a heterogeneous nucleation effect, resulting in formation of composites with lower glass transition temperatures (1–3 °C) and higher stiffness (~15%) [53,54].

Saasse et al. [48] also reported that PLA regranulation can reduce molecular weight due to thermal stress while simultaneously improving homogeneity and crystallisation behaviour [49].

Shortening of polymer chains is a common consequence of thermomechanical stresses during re-granulation. The loss of structural cohesion is related to the fact that shorter chains are less likely to form crystalline bonds, leading to a lower elastic modulus [49,50].

UV irradiation alone markedly affects PLA, with typical losses of 13–30% in Young’s modulus reported [55]. While inert mineral fillers can accelerate photo-oxidation, bio-fillers rich in antioxidants—such as coffee-ground extracts—can slow it by absorbing UV light [55,56]. Coffee grounds indeed contain antioxidative compounds that mitigate UV-induced degradation [57].

A key result of the present work is that no further significant change in modulus occurred in PLA-CG during UV ageing, nor after a second recycling cycle. As Figure 12 illustrates, the modulus varied only slightly, ranging from an 11.6% increase to a 3.5% decrease, with the CG filler reducing scatter to 2.6–13.2%.

Differences between the modulus values reported here and those in other 3D-printing studies can be ascribed to the layer orientation, nozzle diameter, infill density, print temperature, and the specific UV-exposure protocol. Printing parameters are therefore a major influence, alongside the degradation conditions.

Because a lower Young’s modulus signifies reduced resistance to elastic tensile deformation—that is, a less rigid structure—the observed drop may limit the load-bearing capacity of printed components in service [58,59,60]. Similar reductions in stiffness have been reported by several independent investigations [61,62].

It should be noted that the ability of the composite to retain its mechanical properties after multiple recycling cycles is in line with current research trends. These are being pursued not only by academic institutions but also by filament manufacturers who are striving to develop sustainable and recyclable materials with high functionality. The stability of PLA composites under environmental stress conditions (e.g., UV radiation) is therefore a key parameter in evaluating their application potential for the circular economy in practice.

Based on the tests performed, it can be clearly stated that the addition of coffee grounds to PLA has a significant effect on the behaviour of the material both in the initial state and after exposure to UV degradation and subsequent recycling. The preparation of the composite material itself revealed the practical challenges associated with the use of natural filler—particularly the need for thorough pre-treatment, including drying, sieving, and optimisation of dosage with respect to the desired properties of the resulting material.

The size fraction of 0.2–0.3 mm proved to be a suitable choice in terms of the technological requirements of additive manufacturing (FDM 3D printing). This size ensured the required print flow and minimised the risk of clogging of the printer nozzle (nozzle diameter 0.4 mm). However, due to the use of a screen with a mesh size of 0.3 mm, the presence of smaller particles in the final fraction cannot be excluded, which may affect the homogeneity of the mixture and the interaction of the filler with the polymer matrix.

Long-term UV degradation played a significant role in the material behaviour. It was shown that pure PLA after 12 weeks of degradation became highly brittle and lost shape stability, which made its further processing completely impossible. On the other hand, the composite PLA variant with 10% coffee grounds still showed a higher level of processability after 12 weeks, although the mechanical properties slightly deteriorated. This result may suggest that the natural filler materials partly contribute to maintaining the integrity of the composite materials even after prolonged exposure to UV degradation. This may be due to several factors, such as different UV absorption patterns, higher radiation scattering ability in the matrix, or the possible action of the filler as a barrier to moisture penetration [63,64,65,66]. A similar conclusion was reached by other researchers [66], who investigated the use of a filler in the form of a nano-oil, which significantly reduced the rate of photo-oxidation, probably due to its UV blocking properties and a barrier effect that prevented the penetration of oxygen [67]. The literature also indicates that fillers based on metal oxides and carbonates (TiO_2_, CaCO_3_) in the composite significantly increase the resistance of PLA composites to UV radiation, both in terms of appearance and mechanical properties [63].

Recycling itself is also a significant aspect. Although the pure PLA was no longer processable after 12 weeks, the composite material containing the natural filler component still made it possible to create the filament and subsequently develop the test samples after recycling. The mechanical properties of the recycled samples were lower than those of the original (non-degraded) samples but still applicable in the context of less demanding applications. This shows that PLA composite with coffee grounds can be an attractive option not only from an environmental perspective but also in terms of technical feasibility, durability, and the possibility of multiple processing [68].

**Figure 13:**
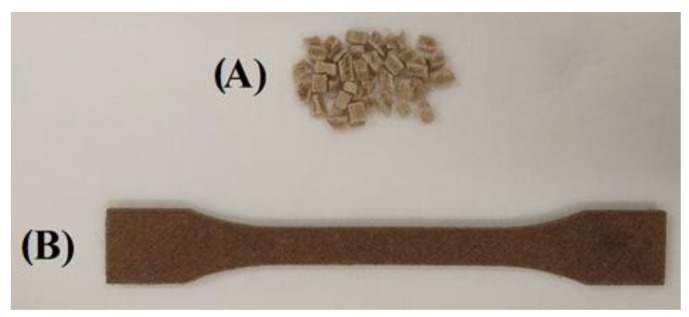
Comparison of PLA_CG material: (**A**) PLA_CG degraded for 12 weeks; (**B**) recycled material A printed into a test specimen using additive technology.

Exposure of the PLA_CG composite material (PLA with 10% coffee grounds) to UV light resulted in visible changes in appearance, mainly fading of the surface of the samples. This phenomenon can be attributed to the photo-oxidation processes that take place on the surface of the polymer matrix, which can also affect the organic components of the natural filler. An interesting finding was that subsequent heat treatment of the degraded samples by extrusion resulted in a partial recovery of the original appearance of the material, as can be seen in Figure 13. The resulting filament after re-extrusion (Figure 13B) and 3D printing showed a similar colour to the initial composite before exposure to UV light. This effect can probably be explained by the homogenization of the colour components during the melting of the material, when the residual pigments from the coffee grounds are redistributed throughout the material.

It is important to note that a significant difference in mechanical properties occurs after a combination of UV degradation and recycling. PLA_CG was able to maintain its integrity in spite of double modification of the material and the resulting weakening. This fact supports the idea that the presence of natural filler reduces the embrittlement of PLA caused by ageing and photodegradation.

**Figure 14:**
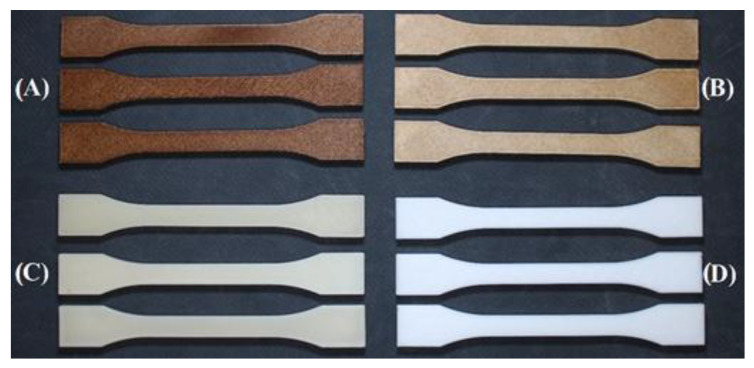
Comparison of optical properties of samples: (**A**) PLA_CG degraded 4 weeks, recycled; (**B**) PLA_CG degraded 4 weeks; (**C**) PLA degraded 4 weeks, recycled; (**D**) PLA degraded 4 weeks.

After visual inspection of the samples, it can be determined that UV degradation causes a change in surface colour. This change is illustrated in Figure 14.

**Material degradation**

The results show that the ability of pure PLA to resist deformation is significantly reduced with increased degradation time. While the non-degraded material showed a mean elongation value of around 5 mm, after 12 weeks of degradation, it dropped dramatically to below 1 mm. This development confirms the known susceptibility of PLA to hydrolysis and degradation in environments with higher humidity and temperature.

The PLA_CG composite material containing CG fillers showed lower but more stable displacement values throughout the experiment. Even after 12 weeks of degradation, its deformation capacity did not drop below 2 mm, indicating better structural integrity and resistance to degradation processes.

**Recycling after degradation**

Significant differences were also observed for the recycled samples. In the case of pure PLA, which was degraded for 4 weeks and then recycled (Recycled 4 weeks), there was a significant increase in the displacement value (approximately 10 mm). This result suggests a loss of structural cohesion and possibly excessive plasticisation or the formation of shorter polymer chains during the recycling process.

On the other hand, PLA_CG maintained similar elongation values after recycling, with no significant deviations, even in the recycled sample degraded for 12 weeks. In contrast, pure PLA could no longer be recycled after 12 weeks of degradation.

The degradation of PLA involves chain scission processes that may shorten the length of individual polymer chains. During subsequent recycling, where the material was remelted and reshaped, these shorter chains may exhibit altered flow and deformation behaviour compared to the original material [69,70]. They slide over each other more easily, without entanglements, and thus allow more plastic deformation before they break. The result is increased ductility of the material—i.e., the ability to elongate significantly before breaking. This may explain the increased displacement values for PLA, recycled after 4 weeks of UV degradation [71].

**Figure 15:**
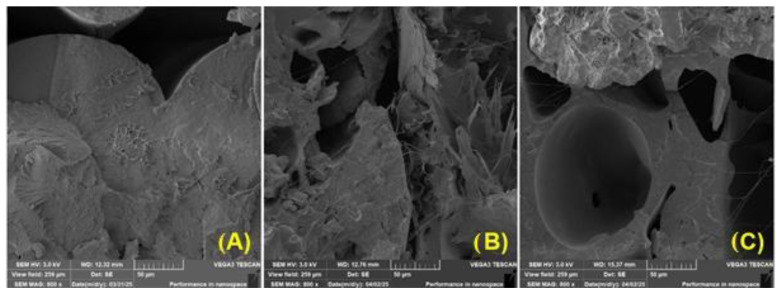
SEM images of fracture surfaces: (**A**) PLA without UV degradation (MAG 800×); (**B**) PLA 4 weeks UV degradation (MAG 800×); (**C**) PLA 12 weeks UV degradation (MAG 800×).

Figure 15 shows scanning electron microscopy (SEM) images of fracture surfaces in PLA samples after various stages of UV degradation, illustrating the morphological changes caused by UV ageing.

Figure 15A shows the fracture surface of a PLA sample without UV degradation. The fracture surface is relatively smooth, with a few cracks and a layered structure typical of a brittle fracture. The surface shows minimal porosity and appears compact overall, with no major signs of damage, corresponding to the properties of non-degraded PLA, which retains its structural integrity and mechanical strength.

In Figure 15B, which shows PLA after 4 weeks of UV degradation, a clear change in surface morphology is already apparent. The fracture surface is significantly rougher and irregularly structured, with numerous microcracks and signs of material fragmentation. These features indicate that polymer chains are degrading, breaking down, and gradually losing cohesion between individual polymer chains. Morphological changes of this type are typical for PLA that has been exposed to UV radiation for a long time.

Figure 15C shows PLA after 12 weeks of UV degradation and exhibits the highest degree of damage. The surface is highly porous, with deep cavities and significant separation of layers. Extensive cracks and cavities are visible, probably caused by advanced photochemical degradation and destruction of macromolecular structures.

The overall appearance of the surface is very unstable and indicates a significant reduction in the mechanical strength of the material. This type of fracture surface is typical of very brittle failure in degraded PLA. These results are consistent with studies by other researchers [72], who described in detail the structural changes in PLA during UV degradation. In particular, some reports [73] directly confirmed the differences in fracture surface morphology depending on the length of exposure. PLA samples without UV irradiation exhibited a smooth, compact surface with pronounced crystallinity and no signs of damage, which corresponds to the characteristics of non-degraded material. After 24 h of UV exposure, a notable breakdown of the polymer chains was observed, accompanied by the formation of microcracks, which may be due to fragmentation of the molecular structure, and partial loss of crystallinity, along with initial signs of macroradical recombinations. After 144 h, PLA is practically amorphous, with a significantly porous structure, deep cracks, and loss of mechanical stability, which corresponds to a severely degraded and brittle material [73,74,75].

**Figure 16:**
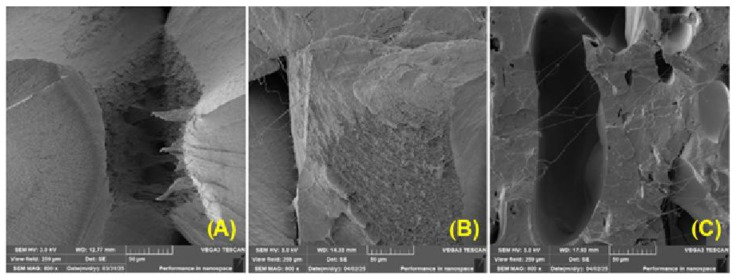
SEM images of fracture surfaces: (**A**) PLA_CG without UV degradation (MAG 800×); (**B**) PLA_CG 4 weeks UV degradation (MAG 800×); (**C**) PLA_CG 12 weeks UV degradation (MAG 800×).

Figure 16 shows the fracture surfaces of composite materials made of PLA mixed with coffee-ground fillers (referred to as PLA_CG) after varying lengths of exposure to UV radiation. Gradual changes in the microstructure of the material due to photodegradation can be observed here. In Figure 16A, i.e., in the case of the PLA_CG sample without UV degradation, the fracture surface is mostly smooth, with characteristics typical of a brittle fracture. A sharp crack with clean and compact edges is visible, indicating a homogeneous structure without significant disruptions. The sample appears cohesive and solid, with no obvious signs of damage.

After 4 weeks of UV degradation, as shown in Figure 16B, there is a significant change in surface morphology. The fracture surface becomes rougher, microcracks appear, and local layering occurs. The surface looks rougher and less cohesive, which shows that the overall stability of the composite is lower.

Figure 16C shows PLA_CG after 12 weeks of UV degradation, when the material was already significantly damaged. The fracture surface is highly porous, with numerous cavities. The overall surface appears significantly damaged and disintegrated, corresponding to a very brittle fracture typical of heavily degraded composite materials with weakened cohesion within the polymer matrix and adhesion between the natural filler and the polymer matrix.

**Figure 17:**
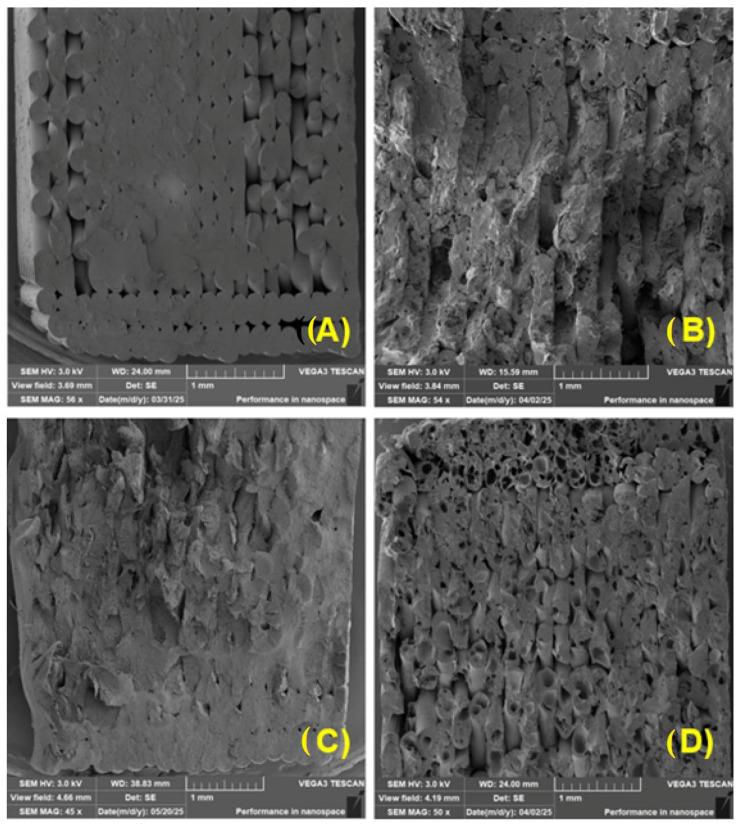
Fracture area of samples: (**A**) PLA without UV degradation (MAG 56×); (**B**) PLA with 4 weeks UV degradation (MAG 54×); (**C**) PLA with 4 weeks UV degradation and recycled (MAG 45×); (**D**) PLA with 12 weeks UV degradation (MAG 50×).

Figure 17 shows increased porosity and delamination of individual layers during 3D printing. The tested materials exhibited increased porosity, probably due to increased degradation of PLA by UV radiation. Similar conclusions were drawn by other studies [76]. Figure 17 shows examples illustrating the layer connections in 3D printing. There is also a noticeable increase in porosity and poor adhesion between individual layers associated with recycling and UV degradation effects.

**Figure 18:**
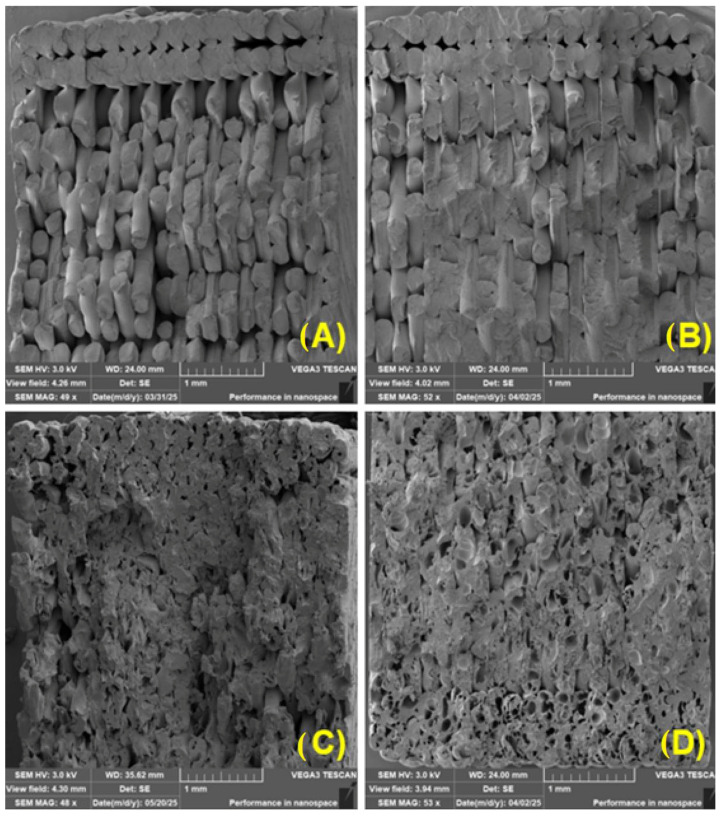
Fracture area of samples: (**A**) PLA_CG without UV degradation (MAG 49×); (**B**) PLA_CG with 4 weeks UV degradation (MAG 52×); (**C**) PLA with 4 weeks UV degradation and recycled (MAG 48×); (**D**) PLA with 12 weeks UV degradation (MAG 53×).

It has been confirmed that poor interlayer bonding may occur when using FDM technology [77]. This deteriorated bonding of individual layers and individual perimeters is also visible in Figure 18, which shows the fracture surfaces of printed samples made of PLA_CG material. 

Figure 18A shows the slicer settings, with two circumferential perimeters visible at the edge of the sample. The printer filled the rest of the sample space at a 45° angle to the circumferential perimeter and at a 90° angle in successive layers.

The coffee-grounds-based filler (CG) significantly reduces UV light permeability due to polyphenolic antioxidants and dark pigments, which significantly reduce oxidative chain scission in PLA [56,78,79]. This enables effective recycling after exposure to intense UV light and a significant change in mechanical properties. It has been reported that coffee-ground extracts increase the UV resistance of PLA-based materials, reducing strength loss by up to 38% [56,80]. It was also been pointed out that even 5% filler has a positive effect on mechanical properties and biodegradability [81]. The displacement (ΔL) results correlate well with the findings of other researchers [82], who tested the mechanical recycling of PLA exposed to UV radiation. The results show that the material rapidly loses its ductility after the first cycle of UV degradation and subsequent regranulation [82].

These findings are significant for enabling effective material recycling when modifying PLA with coffee-ground (CG)-based fillers, as highlighted in the literature [83]. Based on previous research, it is recommended to incorporate any UV protective components directly into the polymer mixture during production. The study demonstrated an insufficient durability of PLA without these modifications in outdoor environments [83,84].

The life cycle assessment (LCA) method was used to compare the impacts of different PLA disposal methods—incineration, landfill, and recycling after UV degradation. Recycling proves favourable in terms of greenhouse gas emissions and energy consumption [85]. The present findings support the development of recycling infrastructure focused on the utilisation of recycled PLA material.

**Figure 19:**
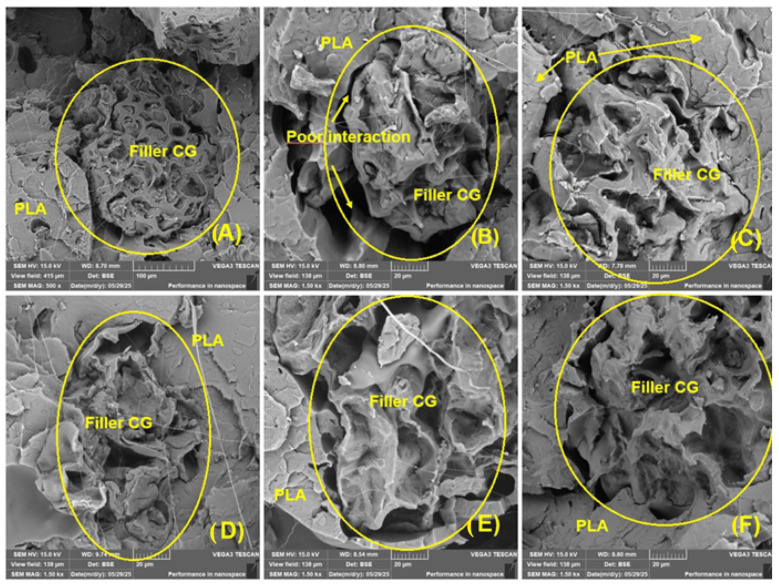
SEM images—interaction of coffee-ground (CG) filler and PLA: (**A**) fracture area of PLA-CG without UV degradation (MAG 500×); (**B**) fracture area of PLA-CG without UV degradation (MAG 1.50k×); (**C**) fracture area of PLA-CG after 4 weeks UV degradation (MAG 1.50k×); (**D**) fracture area of PLA-CG after 4 weeks UV degradation after recycling (MAG 1.50k×); (**E**) fracture area of PLA-CG after 12 weeks UV degradation (MAG 1.50k×); (**F**) fracture area of PLA-CG after 12 weeks UV degradation after recycling (MAG 1.50k×).

Figure 19 shows the interface between the coffee-ground filler and PLA matrix, revealing visible separation and poor cohesion. These features indicate weak interfacial bonding, as shown by voids and partial debonding of filler particles. Despite this, the mechanical performance of the composite (particularly in variant 2) remains only slightly reduced, suggesting that the low filler content and small particle size may limit the influence of poor adhesion at this stage.

A markedly heterogeneous, porous microstructure with local agglomerates of coffee-ground (CG) microparticles is apparent (Figure 17 and Figure 18). Similar limitations of this filler have been noted by other researchers [56,86]. Microparticles recovered from tyre-recycling streams show comparable behaviour: their tendency to agglomerate, irregular geometry, and intrinsic elasticity all help to offset the otherwise weak interaction with an epoxy matrix [87]. In the present study, filler–matrix debonding appeared as gaps of 3.41 ± 3.28 µm (measured on ≥30 SEM micrographs). Although these gaps are relatively small, they nonetheless diminish the adhesive bond between the CG filler and the PLA matrix. An even more critical defect is interlayer delamination arising from insufficient fusion between successive FDM layers; this is also visible in Figure 17 and Figure 18. When interlayer bonding is poor, tensile strength can drop by up to 60% [88,89]. The filler–matrix debonding and interlayer separation act at different length scales, yet jointly they might compromise the structural integrity of the 3D printed material.

This morphological evidence, however, highlights the potential for further material optimisation to enhance filler–matrix interaction.

This negative trend was evident in all tested samples (A–F) in Figure 19. In the literature, poor interaction was revealed at the interface between coffee grounds and PLA after exposure to UV radiation. The occurrence of this adhesive failure at the interface between the matrix and reinforcement can be interpreted as a consequence of poor adhesion between the two phases, also due to their considerable porosity [56].

Although the PLA_CG composite (option 2) showed only a minor reduction in tensile strength and displacement (ΔL) compared to pure PLA (option 1), the SEM images in Figure 19 reveal visible signs of poor interfacial adhesion between the coffee-ground filler and the PLA matrix. This includes the presence of interfacial voids and filler pull-out, which are typical indicators of weak stress transfer capability. Normally, such morphological features would correspond to a more significant decrease in mechanical properties. However, in this study, the effect was limited—likely due to the relatively lower filler content (10 wt.%) and small particle size (0.2–0.3 mm), which allowed uniform dispersion without major agglomerations. At this filler concentration, the mechanical response is still dominated by the PLA matrix, which may mask the local weakening at the filler–matrix interface. This suggests that while morphological features point to suboptimal adhesion, their influence on global tensile behaviour is not very critical in this formulation. Finally, the relatively higher magnification of the SEM micrographs (≈1.5k×) and the heterogeneous, highly porous nature of the filler must be considered. Under these conditions, the observed reduction in interfacial interactions is unlikely to significantly influence the bulk behaviour of the material, whose properties are primarily governed by the manufacturing process and the bonding between successive printed layers. Nevertheless, improving interfacial compatibility through surface treatments or coupling agents may be necessary in future studies, especially for higher filler loadings/concentrations or applications requiring long-term mechanical performance.

Research has shown that recycled PLA exhibits degraded properties, but these can be improved by modifying the fillers. This can challenge the assumption that recycling deteriorates the mechanical properties of PLA [90,91,92].

The results shown in Figure 19A–F do not reveal any clear trend in the differences caused by UV degradation and the recycling process due to the interaction between the filler and the matrix.

Incorporating coffee-ground (CG) filler into PLA retards photo-oxidative degradation. The effect arises from naturally occurring phenolic compounds—principally chlorogenic and caffeic acids together with lignin—that absorb ultraviolet (UV) radiation and act as antioxidants, thereby quenching free radicals and inhibiting polymer-chain scission. Moreover, the dark colour of CG particles, their porous morphology and high specific surface area create a physical barrier that scatters incident UV light and lowers oxygen permeability, further suppressing oxidative degradation of PLA [55,56,57,93].

## 4. Conclusions

The results of this study demonstrated that PLA-based composite materials with added natural fillers, specifically coffee grounds, represent a viable alternative for additive manufacturing applications where ecological properties, recyclability, and stability under environmental stress are required. Compared to pure PLA, the PLA_CG composite exhibited significantly higher mechanical stability after long-term UV degradation and allowed for repeated recycling without significant loss of functionality.

It has been experimentally confirmed that the entire cycle of “printing–degradation–recycling–printing” is technically feasible for composite PLA with natural filler. The presence of coffee grounds has a positive effect on the UV resistance of PLA material, which is particularly beneficial for products intended for outdoor applications. At the same time, it was shown that recycling remains possible even after prolonged degradation, which significantly contributes to extending the product life cycle.

These findings confirm the potential of PLA composites in the context of the circular economy and contribute to the discussions on the sustainable development of additive technologies. Future research could focus on optimising the composition of composites, finding suitable types of bio-waste fillers, and investigating their impact on other functional properties of printed products, such as thermal stability, moisture absorption, and optical characteristics.

The main findings can be summarised as follows:

- Neat PLA exhibited an initial tensile strength of ≈28 MPa (mean value for the as-printed specimens; see Table 3). After 12 weeks of exposure to simulated solar UV (λ = 280–400 nm, irradiance 0.68 W m^−2^), the tensile strength fell below 10 MPa, confirming severe photo-oxidative degradation.
- The PLA_CG composite (PLA + 10 wt. % coffee grounds) started at a lower tensile strength (≈25–27 MPa) but maintained this level after the 12-week UV regime, demonstrating markedly higher UV resistance and structural integrity.
- Recycling performance diverged strongly: neat PLA could not be re-processed after prolonged UV ageing. The melting process failed during filament extrusion, and the re-printed parts were mechanically unstable, whereas PLA_CG was successfully recycled and re-printed without a significant loss of tensile strength.
- The Young’s modulus of neat PLA dropped by ≈20% within the first 4 weeks of UV exposure, and its coefficient of variation increased to ~35%. Although the CG filler lowers the absolute modulus, it stabilises the data scatter (2.6–13.2%) and preserves the long-term stability.

It has been confirmed that PLA_CG composites are better suited to circular manufacturing: they permit repeated printing even after degradation, with a smaller loss in mechanical properties compared to neat PLA. The produced and tested filament combines biodegradability with an extended service life. Using coffee-ground waste as filler supports the circular economy and lowers the environmental burden. The study therefore validates the potential of PLA_CG for sustainable engineering applications using 3D printing technology.

## Figures and Tables

**Table 1:** Variants of sample sets examined.

Option	UV Degradation (Number of Weeks)	Recycled ^2^	Material
1	0	No	PLA
2	0	No	PLA_CG ^1^
3	4	No	PLA
4	4	No	PLA_CG ^1^
5	4	Yes	PLA
6	4	Yes	PLA_CG ^1^
7	12	No	PLA
8	12	No	PLA_CG ^1^
9	12	Yes	PLA
10	12	Yes	PLA_CG ^1^

^1^ PLA_CG: Polylactic acid composite with 10% coffee grounds by weight. ^2^ The recycled material was mechanically crushed, then processed into filaments by extrusion that were subsequently used for additive manufacturing of test bodies using FDM 3D printing.

**Table 2:** Print parameters.

Nozzle size	0.4 mm
Layer height	0.2 mm
Infill density	100%
Infill pattern	Rectilinear
Angle of filling	45°
Number of perimeters	2

**Table 3:** Mechanical test results—static tensile test.

Type of Sample	Option	UV Degradation [Number of Weeks]	Recycled ^1^	σm [MPa]	ΔL [mm]	E [MPa]
PLA	1	0	No	27.73 ± 3.91	5.28 ± 1.32	1797.12 ± 120.70
PLA_CG	2	0	No	27.41 ± 0.53	4.09 ± 0.23	1124.45 ± 50.96
PLA	3	4	No	27.75 ± 5.38	3.37 ± 0.65	1421.86 ± 106.89
PLA_CG	4	4	No	25.36 ± 2.22	3.16 ± 0.25	1088.96 ± 129.04
PLA	5	4	Yes	30.90 ± 1.28	10.74 ± 0.73	1723.64 ± 132.99
PLA_CG	6	4	Yes	25.56 ± 2.38	2.69 ± 0.09	1084.99 ± 143.12
PLA	7	12	No	7.34 ± 1.41	0.50 ± 0.13	1570.65 ± 531.29
PLA_CG	8	12	No	26.95 ± 1.23	2.37 ± 0.18	1254.47 ± 71.36
PLA	9	12	-	-	-	-
PLA_CG	10	12	Yes	25.82 ± 1.01	2.42 ± 0.20	1096.55 ± 28.25

^1^ The recycled material was mechanically crushed, then processed by extrusion to form filaments that were used for additive manufacturing of test bodies using FDM 3D printing.

## Data Availability

The original contributions presented in this study are included in the article. Further inquiries can be directed to the corresponding author.
